# Spatiotemporal analysis of malaria for new sustainable control strategies

**DOI:** 10.1186/s12916-018-1224-2

**Published:** 2018-12-04

**Authors:** Jordi Landier, Stanislas Rebaudet, Renaud Piarroux, Jean Gaudart

**Affiliations:** 1 Aix Marseille Univ, INSERM, IRD, SESSTIM, Sciences Economiques & Sociales de la Santé & Traitement de l’Information Médicale, Marseille, France; 2 APHM, Assistance Publique – Hôpitaux de Marseille, Marseille, France; 3 Hôpital Européen, Marseille, France; 4 Sorbonne Université, INSERM, Institut Pierre-Louis d’Epidémiologie et de Santé Publique, AP-HP, Hôpital Pitié-Salpêtrière, Paris, France; 5 Aix Marseille Univ, APHM, INSERM, IRD, SESSTIM, Hop Timone, BioSTIC, Biostatistic & ICT, Marseille, France

**Keywords:** Sustainable malaria control, spatiotemporal analysis, bottleneck strategies, malaria reservoir, Epidemiological Information System

## Abstract

Malaria transmission is highly heterogeneous through time and space, and mapping of this heterogeneity is necessary to better understand local dynamics. New targeted policies are needed as numerous countries have placed malaria elimination on their public health agenda for 2030. In this context, developing national health information systems and collecting information at sufficiently precise scales (at least at the ‘week’ and ‘village’ scales), is of strategic importance. In a recent study, Macharia et al. relied on extensive prevalence survey data to develop malaria risk maps for Kenya, including uncertainty assessments specifically designed to support decision-making by the National Malaria Control Program. Targeting local persistent transmission or epidemiologic changes is necessary to maintain efficient control, but also to deploy sustainable elimination strategies against identified transmission bottlenecks such as the reservoir of subpatent infections. Such decision-making tools are paramount to allocate resources based on sound scientific evidence and public health priorities.

Please see related article: https://malariajournal.biomedcentral.com/articles/10.1186/s12936-018-2489-9.

## Background

In line with the Sustainable Development Goals numerous countries have placed malaria elimination on their public health agenda for 2030. Understanding the spatial and temporal dynamics of malaria is of utmost importance to deploy sustainable control and elimination interventions. Indeed, resource constraints preclude the generalization of interventions and guide targeted approaches. Following the recommendations of the World Health Organization, maps of malaria transmission heterogeneity are being prepared for national programs to efficiently allocate interventions [1]. Consequently, the development of national epidemiologic information systems is of strategic importance. Nevertheless, the choice of relevant representations of malaria risk heterogeneity and the assessment of uncertainty of results present significant challenges. Indeed, misclassification may result in the lack of service provision to populations in greater need, causing preventable mortality and morbidity. Incorrectly addressing persistent foci of transmission could also jeopardize elimination efforts in some settings. Conversely, resources should not be wasted on interventions in populations who do not require them.

In a recent article, Macharia et al. [2] analyzed the spatiotemporal prevalence of *Plasmodium falciparum* malaria in Kenya based on an extensive collection of 5020 malaria surveys conducted at 3701 communities over 35 years (1980–2015). The authors predicted country-scale maps of annual risk of *P. falciparum* infection for children aged 2–10 years (PfPR_2–10_), using a geostatistical model fitted on a limited number of surveyed parameters, namely age, number of samples, location, and time of survey.

## Stratifying malaria and assessing uncertainty with limited data

Interest in such geostatistical modelling does not stem simply from its ability to produce probability maps, which could also be drawn using country-representative cross-sectional surveys (such as the Malaria Indicator Survey included in the Demographic and Health Surveys program), but also from its inclusion of estimates of the certainty of malaria risk prediction, which can prove very useful for decision-making. Building on limited assumptions and extensive prevalence data, as the authors were able to do in Kenya, is close to ideal, albeit not always possible. The contribution of historical data to the assessment of the current situation deserves attention, considering changes in the diagnostic methods, from microscopy to rapid diagnostic tests (RDTs), as well as changes in malaria epidemiology.

Microscopy is the only consistent laboratory method allowing comparison of cross-sectional survey results over 35 years, as presented by Macharia et al. [2]; however, there are limitations to the use of microscopically defined prevalence as a general indicator of malaria transmission. The interpretation of a given prevalence value, such as ‘PfPR_2–10_’, changes with malaria epidemiology and transmission intensity, and specific human and vector behaviors result in high risks for different population groups. For example, in Southeast Asia, unlike in Kenya, young adult males are at highest risk through occupational exposure to forest-dwelling vectors. In addition, the percentage of asymptomatic subpatent infections undetectable by microscopy and RDT increases with decreasing incidence, which makes estimating the size of the parasite reservoir more difficult in low transmission settings. These subpatent infections could represent more than 80% of carriers in some settings, and they have been shown to contribute to malaria persistence and epidemic recurrence [3–5]. Finally, local changes in the interpretation of prevalence-based indicators can also result from middle- to long-term modifications of the malaria transmission environment (deforestation, irrigation works, changing behavior of vectors, etc.) [6]. Therefore, context-specific prevalence indicators would likely have to be developed to identify subpatent infections.

The generalization of RDTs has led to a drastic increase in the proportion of laboratory-confirmed malaria episodes reported by health systems. Indeed, RDTs require a lower level of equipment and skills, and a much less stringent quality control compared to microscopy. RDT deployment in health centers or at community level across entire countries has significantly improved access to diagnosis and treatment for populations, as well as contributing to improving the precision and reliability of malaria clinical incidence estimates, with the caveat that pfhrp2- and pfhrp3-deleted clones do not reach high prevalence [7].

Consequently, based on data available from public healthcare providers, annual parasite incidence-based stratification remains the recommended strategy. While it sometimes relies on outdated incomplete datasets at a very coarse spatial scale, the continued support and investments to develop national health information systems have resulted in improving quality, completion, and resolution of available incidence datasets. Incidence recorded routinely and continuously at the local level (community health worker, health center, etc.) provides valuable additional spatiotemporal information rarely captured by cross-sectional surveys.

Countries with a reactive surveillance system and nationwide retrospective data already have the capacity to report incidence-based analysis of malaria trends [8, 9]. Without relying on prevalence, incidence maps and models also provide useful information on temporal trends of malaria, matched with uncertainty levels, ultimately guiding elimination programs with relevant information for decision-making, prioritization, and adjustment of on-going interventions. The combination of incidence series with specifically collected prevalence data using sufficiently sensitive tests (polymerase chain reaction or ultrasensitive-RDT) to estimate the size of the asymptomatic reservoir will enable the identification and development of responses targeted to transmission bottlenecks [10].

Other programs [11–16] rely on more complex analysis frameworks, including mathematical simulations, compensating sparse data with hypotheses on the relationship between environmental conditions and risk. The complex relationships between environmental, demographic, and social factors and malaria transmission are difficult to assess accurately, especially when studying historical databases. The increasing availability and quality of national health information data will further minimize the need for underlying assumptions. New layers of data, such as precise environmental factors, parasite genomics, and population mobility, are also becoming available and allow more accurate discrimination of sources from sinks [17]. In all cases, ground-truthing should remain a priority to ensure the validity of the models and of the actions taken upon their outputs.

## Towards real-time surveillance and early warning systems

While the links between incidence and transmission remain complex, incidence data provide a dynamic view of malaria epidemiology in terms of seasonal patterns and yearly variations, and can be collected in a timely manner. A shift from reactive to proactive strategies can also take place when real-time surveillance data is integrated with sentinel sites and weather/climate data in order to produce early warnings [18, 19]. In such information systems, the first challenge is to combine information arising from sources such as health organizations and economic, social, environmental, and population movements. Indeed, the complex transmission dynamics derive from a large range of associated factors. The second challenge is related to the information flow and portfolio of evidence-based and sustainable control strategies. Development of information systems and real-time data should generate retro-information loops, where integrated data is fed back to local-level health professionals (e.g. for inventory management or epidemiological investigations). This approach allows the deployment of locally tailored strategies, such as screening and treatment campaigns, targeted mass drug administration, reactive case detection, contextual community engagements, enhanced vector control, and setting up or reinforcing capacities in a community-based treatment facility in remote villages, to reach elimination in a changing environment.

## Conclusion

Spatiotemporal analysis of malaria dynamics based on epidemiological surveillance systems is needed to collect accurate local information and guide decision-making. As transmission decreases, the heterogeneity of malaria epidemiology increases. In such situations, targeting transmission ‘bottlenecks’, such as addressing residual foci, persisting transmission periods, and parasite reservoir, becomes a priority. In a changing environment, sustainable and adaptive strategies should now be directed from an informed local level.

## References

[1] Bannister-Tyrrell M, Verdonck K, Hausmann-Muela S, Gryseels C, Ribera JM, Grietens KP (2017). Defining micro-epidemiology for malaria elimination: systematic review and meta-analysis. Malaria J.

[2] Macharia PM, Giorgi E, Noor AM, Waqo E, Kiptui R, Okiro EA, Snow RW (2018). Spatio-temporal analysis of Plasmodium falciparum prevalence to understand the past and chart the future of malaria control in Kenya. Malaria J.

[3] Okell LC, Bousema T, Griffin JT, Ouédraogo AL, Ghani AC, Drakeley CJ (2012). Factors determining the occurrence of submicroscopic malaria infections and their relevance for control. Nat Commun.

[4] Coulibaly D, Travassos MA, Tolo Y, Laurens MB, Kone AK, Traore K, Sissoko M, Niangaly A, Diarra I, Daou M, Guindo B, Rebaudet S, Kouriba B, Dessay N, Piarroux R, Plowe CV, Doumbo OK, Thera MA, Gaudart J (2017). Spatio-temporal dynamics of asymptomatic malaria: bridging the gap between annual malaria resurgences in a Sahelian environment. Am J Trop Med Hyg.

[5] Tadesse FG, Slater HC, Chali W, Teelen K, Lanke K, Belachew M, Menberu T, Shumie G, Shitaye G, Okell LC, Graumans W, van Gemert GJ, Kedir S, Tesfaye A, Belachew F, Abebe W, Mamo H, Sauerwein R, Balcha T, Aseffa A, Yewhalaw D, Gadisa E, Drakeley C, Bousema T (2018). The relative contribution of symptomatic and asymptomatic Plasmodium vivax and Plasmodium falciparum infections to the infectious reservoir in a low-endemic setting in Ethiopia. Clin Infect Dis.

[6] Imwong M, Nguyen TN, Tripura R, Peto TJ, Lee SJ, Lwin KM, Suangkanarat P, Jeeyapant A, Vihokhern B, Wongsaen K, Van Hue D, Dong le T, Nguyen TU, Lubell Y, von Seidlein L, Dhorda M, Promnarate C, Snounou G, Malleret B, Rénia L, Keereecharoen L, Singhasivanon P, Sirithiranont P, Chalk J, Nguon C, Hien TT, Day N, White NJ, Dondorp A, Nosten F (2015). The epidemiology of subclinical malaria infections in South-East Asia: findings from cross-sectional surveys in Thailand-Myanmar border areas, Cambodia, and Vietnam. Malar J.

[7] Beshir KB, Sepulveda N, Bharmal J, Robinson A, Mwanguzi J, Busula AO, de Boer JG, Sutherland C, Cunningham J, Hopkins H (2017). Plasmodium falciparum parasites with histidine-rich protein 2 (pfhrp2) and pfhrp3 gene deletions in two endemic regions of Kenya. Sci Rep.

[8] Wangdi K, Canavati SE, Ngo TD, Tran LK, Nguyen TM, Tran DT, Martin NJ, Clements ACA (2018). Analysis of clinical malaria disease patterns and trends in Vietnam 2009–2015. Malaria J.

[9] DePina AJ, Niang EHA, Andrade AJB, Dia AK, Moreira A, Faye O, Seck I (2018). Achievement of malaria pre-elimination in Cape Verde according to the data collected from 2010 to 2016. Malaria J.

[10] Landier J, Parker DM, Thu AM, Lwin KM, Delmas G, Nosten FH (2018). Malaria Elimination Task Force Group. Effect of generalised access to early diagnosis and treatment and targeted mass drug administration on Plasmodium falciparum malaria in Eastern Myanmar: an observational study of a regional elimination programme. Lancet.

[11] Raso G, Schur N, Utzinger J, Koudou BG, Tchicaya ES, Rohner F, N’Goran EK, Silué KD, Matthys B, Assi S, Tanner M, Vounatsou P (2012). Mapping malaria risk among children in Côte d’Ivoire using Bayesian geo-statistical models. Malaria J.

[12] Sturrock HJW, Cohen JM, Keil P, Tatem AJ, Le Menach A, Ntshalintshali NE, Hsiang MS, Gosling RD (2014). Fine-scale malaria risk mapping from routine aggregated case data. Malaria J.

[13] Riedel N, Vounatsou P, Miller JM, Gosoniu L, Chizema-Kawesha E, Mukonka V, Steketee RW (2010). Geographical patterns and predictors of malaria risk in Zambia: Bayesian geostatistical modelling of the 2006 Zambia national malaria indicator survey (ZMIS). Malaria J.

[14] Samadoulougou S, Maheu-Giroux M, Kirakoya-Samadoulougou F, De Keukeleire M, Castro MC, Robert A (2014). Multilevel and geo-statistical modeling of malaria risk in children of Burkina Faso. Parasites Vectors.

[15] Ouedraogo B, Inoue Y, Kambiré A, Sallah K, Dieng S, Tine R, Rouamba T, Herbreteau V, Sawadogo Y, Ouedraogo LSLW, Yaka P, Ouedraogo EK, Dufour JC, Gaudart J (2018). Spatio-temporal dynamic of malaria in Ouagadougou, Burkina Faso, 2011–2015. Malaria J.

[16] Dambach P, Machault V, Lacaux JP, Vignolles C, Sié A, Sauerborn R (2012). Utilization of combined remote sensing techniques to detect environmental variables influencing malaria vector densities in rural West Africa. Int J Health Geogr.

[17] Ihantamalala FA, Herbreteau V, Rakotoarimanana FMJ, Rakotondramanga JM, Cauchemez S, Rahoilijaona B, Pennober G, Buckee CO, Rogier C, Metcalf CJE, Wesolowski A (2018). Estimating sources and sinks of malaria parasites in Madagascar. Nat Commun.

[18] Girond F, Randrianasolo L, Randriamampionona L, Rakotomanana F, Randrianarivelojosia M, Ratsitorahina M, Brou TY, Herbreteau V, Mangeas M, Zigiumugabe S, Hedje J, Rogier C, Piola P (2017). Analysing trends and forecasting malaria epidemics in Madagascar using a sentinel surveillance network: a web-based application. Malaria J.

[19] Kelly GC, Hale E, Donald W, Batarii W, Bugoro H, Nausien J, Smale J, Palmer K, Bobogare A, Taleo G, Vallely A, Tanner, Vestergaard LS, Clements ACA (2013). A high-resolution geospatial surveillance-response system for malaria elimination in Solomon Islands and Vanuatu. Malaria J.

